# Neurotoxicity Assessment of Four Different Pesticides Using In Vitro Enzymatic Inhibition Assays

**DOI:** 10.3390/toxics10080448

**Published:** 2022-08-03

**Authors:** Carlos Martins-Gomes, Tiago E. Coutinho, Tânia L. Silva, Tatiana Andreani, Amélia M. Silva

**Affiliations:** 1 Department of Biology and Environment, School of Life Sciences and Environment, University of Trás-os-Montes e Alto Douro (UTAD), Quinta de Prados, 5001-801 Vila Real, Portugal; camgomes@utad.pt (C.M.-G.); tecoutinho@utad.pt (T.E.C.); tanialfs10@gmail.com (T.L.S.); 2 Center for Research and Technology of Agro-Environmental and Biological Sciences (CITAB-UTAD), Quinta de Prados, 5001-801 Vila Real, Portugal; tatiana.andreani@fc.up.pt; 3 GreenUPorto—Sustainable Agrifood Production Research Centre & Department of Biology, Faculty of Sciences of the University of Porto, Rua do Campo Alegre s/n, 4169-007 Porto, Portugal

**Keywords:** glyphosate, imidacloprid, imazalil, lambda-cyhalothrin, acetylcholinesterase, butyrylcholinesterase, tyrosinase, neurotoxicity

## Abstract

Pesticides affect different organs and tissues according to their bioavailability, chemical properties and further molecular interactions. In animal models exposed to several classes of pesticides, neurotoxic effects have been described, including the reduction of acetylcholinesterase activity in tissue homogenates. However, in homogenates, the reduction in enzymatic activity may also result from lower enzymatic expression and not only from enzymatic inhibition. Thus, in this work, we aimed to investigate the neurotoxic potential of four distinct pesticides: glyphosate (herbicide), imazalil (fungicide), imidacloprid (neonicotinoid insecticide) and lambda-cyhalothrin (pyrethroid insecticide), by assessing their inhibitory effect on the activity of acetylcholinesterase (AChE), butyrylcholinesterase (BChE) and tyrosinase, by using direct in vitro enzymatic inhibition methods. All pesticides dose-dependently inhibited AChE activity, with an inhibition of 11 ± 2% for glyphosate, 48 ± 2% for imidacloprid, 49 ± 3% for imazalil and 50 ± 3% for lambda-cyhalothrin, at 1 mM. Only imazalil inhibited BChE. Imazalil induced dose-dependent inhibition of BChE with identical pattern as that observed for AChE; however, for lower concentrations (up to 500 μM), imazalil showed higher specificity for AChE, and for higher concentrations, the same specificity was found. Imazalil, at 1 mM, inhibited the activity of BChE by 49 ± 1%. None of the pesticides, up to 1 mM, inhibited tyrosinase activity. In conclusion, the herbicide glyphosate shows specificity for AChE but low inhibitory capacity, the insecticides imidacloprid and λ-cyhalothrin present selective AChE inhibition, while the fungicide IMZ is a broad-spectrum cholinesterase inhibitor capable of inhibiting AChE and BChE in an equal manner. Among these pesticides, the insecticides and the fungicide are the ones with higher neurotoxic potential.

## 1. Introduction

**Figure 1:**
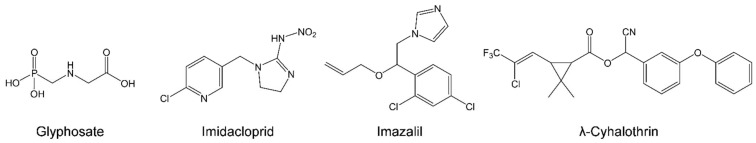
Chemical structure of the herbicide glyphosate, the fungicide imazalil and the insecticides imidacloprid and λ-cyhalothrin.

The use of pesticides in agricultural activities is widespread worldwide for the control of undesired species of plants, insects, and fungi. With the exponential growth in pesticide use, the environmental, animal and human exposure to these chemicals increases proportionally [1,2,3]. While pesticides are a valuable tool to increase yield, profit and, in some cases, food quality, they also arise as toxic components to non-target species, despite often being labeled as non-toxic or presenting minimal risk [4]. Pesticide exposure for the general population is mainly due to contaminated food products and water, whose long-term exposure has an impact on human health, particularly on the nervous system. Various pesticides have been shown to bioaccumulate in the central nervous system (CNS) and induce neurotoxicity. For example, an increased risk of developing Parkinson’s and Alzheimer’s diseases has been linked to pesticide exposure [5,6,7]. In the case of Alzheimer’s disease, the mechanisms behind the pesticide effect are most likely connected to oxidative stress induction, hyper-phosphorylation of tau protein and interaction/disruption of amyloid-beta protein homeostasis [6]. In Wistar rats, glyphosate (GLY; Figure 1) was shown to dose-dependently alter the brain levels of serotonin, dopamine and norepinephrine. These changes were dependent on the brain region, which is an indication that the herbicide is able to permeate the blood-brain barrier and supposedly accumulate in the brain, causing neurotoxicity [8]. Additionally, GLY-based herbicides modulated amino acid metabolism at the brain level in Wistar rats, elevating the levels of excitatory neurotransmitters aspartate and glutamate, indicating the neurotoxic effect of GLY-based herbicides [9]. Many toxicological studies reporting the toxicity of GLY use GLY-based herbicides, such as Roundup, and not the pure compound, but recent studies highlight that the surfactant used in GLY-based herbicides (POEA, polyethoxylated tallow amine) is the main cause of toxicity [3,10]. Indeed, in vivo studies using a fish model showed about 10-fold higher toxicity when using GLY-based herbicides compared to the GLY standard [10]. Additionally, GLY-based herbicide and aminomethylphosphonic acid (AMPA; glyphosate metabolite) induced pronounced cytotoxicity and neurotoxicity in SH-SY5Y cells (human neuroblastoma) through increasing intracellular reactive oxygen species (ROS) and by altering the expression of genes related to neuronal development, apoptosis and autophagy [11]. For fungicides such as imazalil (IMZ; Figure 1), also referred to as enilconazole, it produced neurotoxicity in larval zebrafish, which was observed by changes in locomotor behavior being attributed to a decrease in acetylcholinesterase (AChE) expression and activity, to a decrease in dopamine content, and modulation of neurotoxicity related genes [12]. IMZ induced oxidative stress in PC12 cells (rat pheochromocytoma cell line), a model often used in neurotoxicological studies [13]. More relevant to neurotoxicity are insecticides, as they often target insects’ nervous systems, such as imidacloprid (IMD, a neonicotinoid) and λ-cyhalothrin (LCT, a pyrethroid) (Figure 1). IMD targets nicotinic acetylcholine receptors (nAChR) in insects with theoretical minimal toxicity to vertebrates [14]. However, studies conducted in rats showed that IMD reduced the levels of the neurotransmitters: serotonin, gamma-aminobutyric acid (GABA) and dopamine [15] and also the activity of AChE in the brain and plasma [16]. IMD also induced oxidative damage in the rats’ brain while reducing the expression of antioxidant defense enzymes, supporting its neurotoxicity [15,17,18]. Findings concerning LCT, whose primary target is the voltage-sensitive sodium channels of insects [19], indicated a strong effect on rat brain oxidative stress by reducing the activity of antioxidant enzymes and of glutathione content resulting in increased oxidative damage. LCT also reduced rat’s brain AChE activity [20] and AChE expression in the hippocampus [21]. Additionally, *Channa punctatus* (snakehead fish) exposed for 96 h to sub-lethal doses of CLT showed decreased activity of AChE in the brain, gills and muscle [22].

As seen above, although not being the structural target, AChE seems to be a target for many pesticides, and due to the primary role of AChE in neuronal physiology, i.e., in terminating neuronal signaling between synapses and transmission, its homeostasis disturbance leads to several diseases, namely those involving memory and neuromotor function, such as Alzheimer’s disease [23] and other disorders involving dementia [24]. Additionally, in peripheral tissues, such as erythrocytes, AChE disturbance is implicated in several pathologies and is a biomarker of Hirschsprung’s disease and of inflammation [25].

In the case of AChE inhibition in animals, accumulation of acetylcholine affects nervous impulse transmission, with symptoms that include blood vessels vasodilation, decreased heart rate, vomiting, sweating, intestinal cramps, muscle contraction, eye pupil constriction and, ultimately, cardiac failure, paralysis and death. Pesticide-contaminated water has been reported to affect AChE in fish species by reducing their mobility and spatial awareness, reducing both the ability to feed and evade predators [26,27].

Carbamates and organophosphates are within the group of pesticides with higher cholinesterase inhibition; AChE is considered a molecular target for these compounds. For this reason, cholinesterases have been proposed as biomarkers to assess the environmental risk of these pesticides [28]. Various methods have been applied to organophosphates detection in environmental and food samples using AChE, constructing biosensors that present significant advantages such as fast results detection and which are being improved using micro and nanotechnology [29]. Advanced systems such as disposable membrane chips have been developed based on organophosphate-dependent AChE inhibition. Nevertheless, other enzymes may be potential targets for pesticides, such as other cholinesterases, e.g., butyrylcholinesterase (BChE), or tyrosinase, the latter is involved in dopamine metabolism and potentially involved in Parkinson’s disease [30]. BChE, also known as pseudocholinesterase or nonspecific cholinesterase, hydrolyzes esters of choline, including butyrylcholine, propionylcholine and acetylcholine; it hydrolyses butyrylcholine four times faster than acetylcholine and is also inhibited by organophosphate and carbamate pesticides [31]. Since butyrylcholine does not occur in the body naturally, it is not a physiological substrate for cholinesterases [31,32]. BChE is produced in the liver and is then disturbed by other tissues via plasma [23,32,33], being present at the neuromuscular junction, synapses, blood and other tissues [34,35].

AChE and BChE present 65% similarity in their structure [32]. In the brain, BChE is thought to represent 10% of total cholinesterase activity, performing support to AChE activity. These enzymes have different activity rates depending on substrate concentration: at low acetylcholine concentrations, AChE presents higher efficiency, whereas BChE has higher performance at high concentrations of the neurotransmitter, as AChE is inhibited by the substrate [32]. Both enzymes have been seen as therapeutic targets for the treatment of Alzheimer’s disease and diabetes mellitus neurological complications [32]. On the other hand, unwanted inhibition, such as that made by toxicological agents, has been well studied; an example of those studies is the action of pesticides in healthy individuals, in which the inhibition of cholinesterases impairs motor and cognitive functions.

Among the known AChE isoforms found in various vertebrates, AChE isoforms obtained from electric eel (*Electrophorus electricus*) or from *Torpedo* genus (electric ray) present high similarity to the human isoforms and thus being used in in vitro screening assays [36]. Electric ray’s AChE active site presents high similarity to human’s AChE, being an established form to be used in pesticide-induced inhibition of this enzyme [37]. Electric ray AChE active site differs in one amino acid when compared to human AChE, a change from Tyr337 to Phe330, which is reported as not significant [38]. This differs from other models used in neurotoxicity, such as *Drosophila melanogaster*, which present a higher number of structural differences that affect the electrostatic properties of the enzyme [39]. Regarding BChE, the common isoform used in vitro is obtained from equine serum, shares more than 90% similarity with the human isoform, lacking a cysteine residue and an *N*-glycosylation site [40]. Concerning pesticide effect on AChE activity, it is known that organophosphates, whose primary target in neurotoxicity is AChE, are performed by interacting with Ser203, a key amino acid in acetylcholine hydrolysis [37]. Nevertheless, there is considerably little information regarding the neurotoxicity induced by other classes of pesticides, namely their ability to inhibit AChE or BChE.

Tyrosinase is a key enzyme in the melanin synthesis in skin and hair, but it is also involved in the neuromelanin synthesis, which is used to identify susceptible neurons in Parkinson’s disease, and also generates dopamine-quinones [41], which are involved in oxidative stress. Although neuromelalin has been reported as neurotoxic, its neuroprotective effect was attributed to it sequestering metal ions that can be highly cytotoxic [41].

Therefore, it is clear that specific groups of pesticides can specifically inhibit AChE, but fewer studies have considered different cholinesterases or other enzymes relevant to neurotoxicity/neuroprotection such as tyrosinase in assays with easier correlation to human toxicity. Furthermore, some studies reported inhibition of AChE activity in tissue homogenates obtained from various animal models after exposure to different pesticides, as mentioned above. However, when evaluating AChE activity in homogenates, the results of reduced enzymatic activity may not only be related to direct enzyme inhibition but may also be the result of decreased enzyme levels, as pesticides can modulate gene expression. Thus, the main aim of this research was to evaluate the direct in vitro inhibitory effect of an herbicide, glyphosate, a fungicide, imazalil, and two non-carbamates and non-organophosphate insecticides, imidacloprid and λ-cyhalothrin, in AChE, BChE and tyrosinase. In addition, a comparison between the specificity of these pesticides to these enzymes was performed, aiming to predict neurotoxicity or to develop new sensor devices based on the specific interaction of pesticides with key enzyme systems.

## 2. Materials and Methods

### 2.1. Materials and Reagents

Glyphosate (PESTANAL^®^, analytical standard; CAS N. 1071-83-6; MW: 169.07), Imidacloprid (PESTANAL^®^, analytical standard; CAS N. 138261-41-3; MW: 255.66), Imazalil (PESTANAL^®^, analytical standard; CAS N. 35554-44-0; MW: 297.18), lamba-cyhalothrin (PESTANAL^®^, analytical standard; CAS N. 91465-08-6: MW: 449.8), acetylcholinesterase from *Electrophorus electricus* (CAS N. 9000-81-1; E.C. 3.1.1.7), butyrylcholinesterase from equine serum (CAS N. 9001-08-5; E.C. 3.1.1.8), tyrosinase from mushroom (CAS N. 902-10-2; E.C. 1.14.18.1), acetylthiocholine iodide (CAS N. 1866-15-5; MW: 289.18), butyrylthiocholine iodide (CAS N. 1866-16-6; MW: 317.23), 5,5′-dithiobis(2-nitrobenzoic acid) (DTNB; CAS N. 69-78-3; MW: 396.35), 3,4-dihydroxy-*L*-phenylalanine (CAS N. 59-92-7; MW: 197.19) and kojic acid (CAS N. 501-30-4; MW: 142.11) were purchased from Merck (Darmstadt, Germany). All other reagents and salts were of analytical grade and were also obtained from Merck (Darmstadt, Germany).

### 2.2. Enzyme Inhibition Assays

Stock solution of GLY (at 40 mM) was made in distilled water, and the stock solutions of IMD, IMZ and LCT (all at 20 mM) were made in DMSO. All further dilutions were made in ultrapure distilled water. DMSO percentage at the highest tested concentration was 5%, and an enzymatic dose–response inhibition of DMSO was made in all assays. When inhibition occurred, the % of inhibition due to DMSO was subtracted from the respective sample.

#### 2.2.1. Cholinesterase Inhibition Assay

Pesticide-induced inhibition of cholinesterases was performed for AChE and BChE using Elman’s method [42]. Elman´s method is based on the reaction between the thiol group of thiocholine and DTNB (5,5′-dithiobis (2-nitrobenzoic acid) ion forming 5-thio(2-nitrobenzoic acid) that presents yellow color and thus can be quantified by spectrophotometry. Thiocholine is a product of acetylthiocholine and butyrylthiocholine hydrolysis by AChE and BChE, respectively. Thus, the activity of AChE and BChE is directly proportional to the formation of 5-thio(2-nitrobenzoic acid).

The enzyme inhibition assays were performed in 96-well plates as described by Taghouti, et al. [43]. For the AChE inhibition assay, to 50 µL of GLY, IMD, IMZ or LCT test solutions (at 10 to 1000 µM) were added: 125 μL of 0.3 mM DTNB [5,5′-dithiobis (2-nitrobenzoic acid); prepared in 50 mM Tris–HCl, pH 8] and 25 μL of 1.5 mM ATCI (acetylthiocholine iodide; prepared in 20 mM Tris-HCl, pH 7.5), and the mixture was incubated for 2 min, at room temperature, in the dark. Then, 25 μL of 0.026 U/mL AChE (prepared in 20 mM Tris-HCl, pH 7.5) was added to each well, followed by a 20 min incubation. The absorbance (Abs) was then measured at 405 nm using a microplate spectrophotometer (Multiskan SkyHigh from Thermo-Fisher Scientific; Waltham, MA, USA). Each assay was performed in triplicates, and *n* = 3 independent assays were made.

For BChE inhibition, an identical experimental procedure was performed (same buffers and incubation conditions), but using as enzyme substrate BCTI (butyrylthiocholine iodide; prepared in 20 mM Tris-HCl, pH 7.5) and using BChE (25 μL; 0.026 U/mL; prepared in 20 mM Tris-HCl, pH 7.5) as enzyme. In both assays, distilled water was used as negative control. The enzyme inhibition (%) was calculated according to Equation (1):

(1) $$\mathrm{Inhibition}\;\left(\%\right)=\left(\frac{\mathrm{Abs}\;\mathrm{control}-\mathrm{Abs}\;\mathrm{sample}}{\mathrm{Abs}\;\mathrm{control}}\right)\times 100$$

#### 2.2.2. Tyrosinase Inhibition Assay

Tyrosinase inhibition by selected pesticides was performed using the L-DOPA (3,4-dihydroxy-L-phenylalanine) enzymatic oxidation assay [43]. Tyrosinase catalyzes the oxidation of L-DOPA into dopaquinone and then into dopachrome, which presents a brown color and allows a spectrophotometric quantified [44]. In each well, to 25 µL of GLY, IMD, IMZ or LCT test solutions (10–1000 µM) were added: 80 μL of phosphate buffer (50 mM; pH 6.8) and 40 μL of 2.5 mM L-DOPA (prepared in 50 mM phosphate buffer at pH 6.8). The mixture was incubated for 2 min (37 °C; in the dark; using a benchtop incubator; Labnet-Corning, Corning, NY, USA). Then, 40 μL of tyrosinase solution (40 U/mL; prepared in 50 mM phosphate buffer at pH 6.5) was added to initiate the reaction, and the mixture was incubated for 10 min (37 °C; in the dark). The absorbance (Abs) was then measured at 492 nm using a microplate spectrophotometer (Multiskan SkyHigh from Thermo-Fisher Scientific; Waltham, MA, USA). Distilled water was used as negative control and kojic acid as positive control. The inhibition (%) was calculated according to Equation (1).

### 2.3. Data and Statistical Analysis

The data are reported as mean ± SD (*n* = 3 independent experiments, each one in triplicates). Statistical significance of differences between samples and the control, or between different concentrations, was assessed by the analysis of variance (ANOVA) with Tukey’s multiple comparison test (α = 0.05), using the tools of GraphPad Prism version 7 (GraphPad Software Inc., San Diego, CA, USA). Statistical significance between the results of the two enzymes was assessed by two-way ANOVA with Sidak´s multiple comparison test (α = 0.05), using the tools of GraphPad Prism version 7. Results from ANOVA analysis, namely degrees of freedom, F-value and *p*-value, are presented in Supplementary Table S1.

## 3. Results and Discussion

### 3.1. Pesticide-Induced Cholinesterase Inhibition

Cholinesterases (ChE) enzyme family are a group of hydrolases that break down esters of choline, such as acetylcholine, butyrylcholine or propionylcholine [31]. Acetylcholine (ACh) is a neurotransmitter with many functions in the brain as well as in other organs, intervening in many physiological processes, being the main neurotransmitter at the neuromuscular junction, but also intervening in the autonomic nervous system and others [45]. ACh is released from pre-synaptic neurons into the synaptic cleft (or neuromuscular junction), diffusing and then binding to the acetylcholine receptors in the post-synaptic cells. Cholinergic signaling is also regulated by cholinesterases, which hydrolyze the neurotransmitter into choline and acetate (ACh hydrolysis), thus terminating the transmission at cholinergic synapses [32,33]. Due to the relevance of cholinergic signaling in several physiological targets and due to the high exposure to different classes of pesticides, either from environmental or food exposure, it is necessary to assess the effect of the various pesticides on cholinesterase activity.

In this study, we analyzed the anti-cholinesterase activity of glyphosate (GLY), imazalil (IMZ), imidacloprid (IMD) and lambda-cyhalothrin (LCT), using in vitro inhibition assays with the potential to be extrapolated to human exposure. Concerning human exposure to these pesticides, limited information is available unless to GLY. For example, after accidental exposure to GLY, a concentration of ~0.52 mM (89 µg/mL) was found in human serum [46]. Considering GLY exposure through food products, values between 2954 µg/day (i.e., 17.47 µmol/day) and 3142 µg/day (18.59 µmol/day) were reported for healthy diets [47], and in cereals values reached 230 mg/kg (i.e., 1.36 mmol) [48]. Regarding IMZ, concentrations up to 10 mg/kg were found in post-harvest citrus fruit [49]. Concerning IMD, this insecticide was quantified in many food products, and a daily intake of IMD was estimated to be 0.004–0.131 µg/kg body weight [50]. According to cell-based assays, besides dose-dependent toxicity, cytotoxicity is also dependent on the pesticide, the cell type and exposure time [51,52]. In order to compare the effects of the four pesticides, the same range of concentrations (up to 1 mM) was used in the experiments.

**Figure 2:**
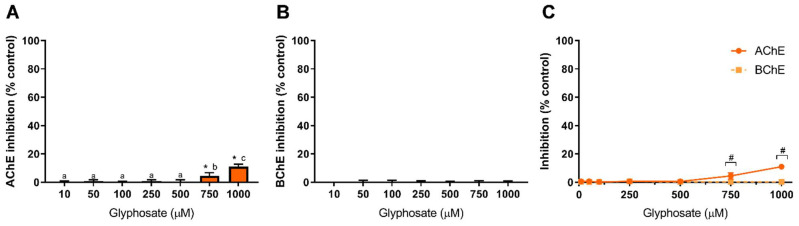
Glyphosate-induced inhibition of AChE (**A**), BChE (**B**) and comparison of cholinesterases affinity (**C**). Values are expressed as mean ± SD. Significant statistical differences between samples and the respective control are denoted by “*” when *p* < 0.05 (**A**,**B**), and statistical differences between enzymes at the same concentration are denoted by “#” when *p* < 0.05 (**C**). Different letters were used to denote significant differences between concentrations when *p* < 0.05 (**A**). Statistical analysis details are described in methods (Section 2.3) and in Table S1.

Figure 2 shows the results obtained for GLY-induced inhibition of AChE and BChE. The herbicide GLY shows a low capacity to inhibit AChE as only at the higher tested concentrations inhibition is observed, being 4.5% and 11% at 750 µM and 1000 µM, respectively (Figure 2A). Concerning BChE (Figure 2B), GLY was not able to inhibit the enzyme at concentrations up to 1 mM. From these data, GLY shows a higher affinity to AChE, as evidenced in Figure 2C; however, statistical differences are only observed at 750 µM and 1000 µM (*p* < 0.05), as depicted. The available literature concerning AChE inhibition by GLY presents very distinct results. Samanta et al. Samanta, et al. [53] reported that in two fish species (*Anabas testudineus* and *Heteropneustes fossilis*), a glyphosate-based herbicide increased AChE activity in the brain, muscle and spinal cord, while a study by Braz-Mota et al. Braz-Mota, et al. [54] reported that a fish species (*Colossoma macropomum)* exposed to Roundup^®^ (containing glyphosate at 360 g/L) presented a reduced AChE activity. Similar findings were reported in other fish species (*Leporinus obtusidens* and *Cyprinus carpio*), also using Roundup^®^, with an observed decrease in AChE activity [55,56]. Nevertheless, the contribution of the excipients and adjuvants present in glyphosate-based herbicides to the observed toxicity has been highly addressed before [3], which is evidenced by variation in LC_50_ values between glyphosate and glyphosate-based formulations [10], thus being difficult to correlate the data available in the literature to a GLY-dependent inhibition of AChE as the action of GLY-based formulations is different from the action o GLY standard molecule. As shown in Figure 2, pure GLY is a weak inhibitor of AChE and has no effect on BChE at concentrations up to 1 mM.

Using human erythrocytes exposed to GLY for 1 h, inhibition of AChE activity by 13% at 0.5 mM GLY was observed, but at 5 mM, the inhibition only increased to 17.6% [57], a low inhibition rate as that here reported. El-Demerdash et al. El-Demerdash, et al. [58] reported an IC_50_ value of 714.3 mM (~120 g/L) to human serum AChE exposed to GLY; a concentration significantly higher than the ones here tested and far above GLY concentrations found in human serum [3]. Concerning BChE, a case study involving a patient that ingested a GLY-based herbicide revealed a decrease in serum levels of BChE [59], while in an assay using a freshwater fish species (*Labeo rohita*) exposed to Roundup^®^, an increase in BChE level was observed and correlated to a potential detoxification activity [60]. Other species, such as toads, have been shown to present inhibition of BChE activity when exposed to GLY-based herbicides [61]. This reveals the high heterogeneity of results, most likely derived from different toxicities among vertebrates and from the potential effect of other compounds present in commercial formulations containing GLY (such as Roundup^®^). Therefore, it is necessary to evaluate the toxicity of pure GLY (standard compound) using assays that allow a good correlation of its effect on cholinesterase activity and neurotoxicity.

**Figure 3:**
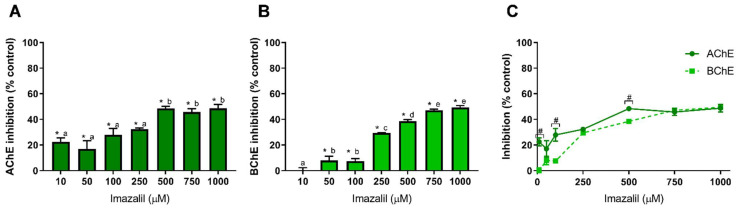
Imazalil-induced inhibition of AChE (**A**) and BChE (**B**), and comparison of cholinesterases affinity (**C**). Values are expressed as mean ± SD. Significant statistical differences between samples and the respective control are denoted by “*” when *p* < 0.05 (**A**,**B**), and statistical differences between enzymes at the same concentration are denoted by “#” when *p* < 0.05 (**C**). Different letters were used to denote significant differences between concentrations when *p* < 0.05 (**A**,**B**). Statistical analysis details are described in methods (Section 2.3) and in Table S1.

The other studied pesticide was the fungicide imazalil. Figure 3 presents IMZ-dependent inhibition of AChE (Figure 3A) and of BChE (Figure 3B), together with the effect on both cholinesterases aiming to assess IMZ specific effect (Figure 3C).

As observed in Figure 3, IMZ induced the most distinguished inhibition pattern for cholinesterases, being the only pesticide analyzed in this study that inhibited both enzymes, AChE and BChE. At 500 µM, IMZ inhibited AChE activity by about 48%, being this the higher inhibition value obtained (Figure 3A). As observed, IMZ concentrations above 500 µM and up to 1 mM did not increase the inhibition of AChE (*p* > 0.05, comparison between concentrations). Nevertheless, it is worth mentioning that concentrations as low as 10 µM produced 22.5% inhibition of AChE activity (*p* < 0.05), thus demonstrating the high neurotoxic potential of this fungicide (Figure 3A). Regarding BChE (Figure 3B), IMZ induced dose-dependent inhibition. Contrarily to AChE, BChE was not inhibited at the lower IMZ concentration, being the inhibitory extent only comparable to IMZ at 250 µM or higher (Figure 3C). Concentrations of IMZ equal to or above 750 µM produced a similar inhibitory effect for both cholinesterases (Figure 3C), revealing a similar affinity for both enzymes. Regardless of the conformational similarities between the two cholinesterases, their active site presents significant differences in size and micro-environment that influence the binding of inhibitors [62]. Thus, it is likely that IMZ inhibition is not performed in the active site, although further studies should be performed to clarify the mechanism behind IMZ-induced inhibition.

Using larval stage zebrafish, Jin et al. Jin, et al. [12] observed that IMZ induced a decrease in AChE activity. Literature analysis revealed that, so far, the inhibitory effect of IMZ in AChE and BChE activity with relevance to human exposure has not been addressed. Thus, to the best of our knowledge, we here report for the first time the neurotoxic potential of the fungicide imazalil in a cholinesterase-dependent manner.

Additionally, and as discussed above, cholinesterases can be useful tools to develop biosensors for ecotoxicological assessment. Unlike the other pesticides analyzed here, IMZ presents affinity to BChE, which can be considered as a potential molecule to use in biosensors development to detect this compound and discriminate between other pesticides. However, additional studies are needed using a wider range of pesticides to access BChE-specific inhibition relative to other common pesticides.

**Figure 4:**
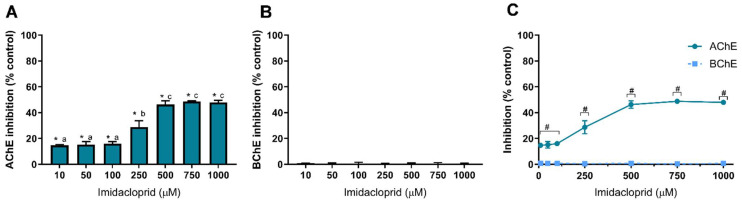
Imidacloprid-induced inhibition of AChE (**A**) and BChE (**B**), and comparison of cholinesterases affinity (**C**). Values are expressed as mean ± SD. Significant statistical differences between samples and the respective control are denoted by “*” (**A**,**B**) when *p* < 0.05, and differences between enzymes at the same concentration are denoted by “#” when *p* < 0.05 (**C**). Different letters were used to denote significant differences between concentrations when *p* < 0.05 (**A**). Statistical analysis details are described in methods (Section 2.3) and in Table S1.

**Figure 5:**
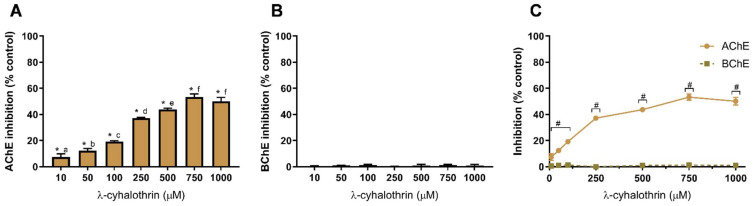
λ-cyhalothrin-induced inhibition of AChE (**A**) and BChE (**B**), and comparison of cholinesterases affinity (**C**). Values are expressed as mean ± SD. Significant statistical differences between samples and the control are denoted by “*” when *p* < 0.05 (**A**,**B**), and differences between enzymes at the same concentration are denoted by “#” when *p* < 0.05 (**C**). Different letters are used to denote significant differences between concentrations when *p* < 0.05 (**A**). Statistical analysis details are described in methods (Section 2.3) and in Table S1.

In this study, we also used two insecticides, IMD and LCT, to assess their inhibitory effect against AChE and BChE. Results of cholinesterase inhibition by IMD are shown in Figure 4 and of LCT are shown in Figure 5. Similar to GLY, IMD only inhibited the activity of AChE (Figure 4A). However, the dose-dependent inhibition pattern of IMD as well as the degree of inhibition is identical to IMZ where 500 μM induces the higher inhibition. As observed, IMD did not inhibit the activity of BChE at concentrations up to 1 mM (Figure 4B), as inhibition values were similar to control (*p* > 0.05), as depicted.

These results reveal that IMD has a higher affinity to AChE, which may potentially be linked to the differences between the two enzymes’ active sites. As observed to IMZ, IMD-induced inhibition reaches its maximum at 500 µM, being the inhibition about to 50% of control, and from 500 µM up to 1 mM, identical inhibition was obtained (*p* > 0.05). At low concentrations (10 µM), IMD is potentially neurotoxic through AChE inhibition (~15% inhibition; Figure 4A). AChE and BChE modulatory effect of IMD was reported for Wistar rats exposed for 28 days to low doses of IMD (up to 2.25 mg/kg b.w./day); however, no statistically significant changes were observed for the activity of both cholinesterases present in plasma or brain [63]. Rainbow trout (*Oncorhynchus mykiss*) exposed to IMD (up to 20 mg/L) for 21 days showed brain AChE inhibition, with 35% inhibition at the highest concentration [64], while a study using shrimp (*Penaeus monodon*) reported elevated AChE activity, in all examined tissues, after 21 days of exposure to IMD [65]. As described above, the IMD effect seems to be organism-dependent. IMD intoxication causes a response similar to nicotine, as neonicotinoids are agonists of the nicotinic ACh receptors (presumably with high selectivity against insect receptors) with toxicity cases presenting vomits, hypertension and tachycardia, evolving cardiac arrest, respiratory failure and death [66]. IMD has been reported to be found in human fluid samples such as blood [67] or urine [68], but also in food products such as cocoa [69] or wine [70]. In the latter, IMD was observed to resist the vinification process, being present in the final product. For these reasons, it is critical to understand the molecular targets and the mechanisms behind IMD neurotoxicity and to find biomarkers for IMD exposure, such as AChE.

Figure 5 shows the effect of the other insecticide, LCT, against AChE (Figure 5A) and BChE (Figure 5B).

The pyrethroid insecticide LCT produced a similar pattern of AChE and BChE inhibition (Figure 5) as the other tested insecticide (IMD, Figure 4). Selective inhibition of AChE was observed when compared to BChE (Figure 5C). Indeed, LCT did not inhibit BChE as inhibition values are not different from the control (*p* > 0.05), as depicted in Figure 5B. LCT produced, on average, the highest inhibition of AChE, being the enzyme inhibition at 750 µM of 53 ± 2%; higher than IMZ (*p* < 0.05) but not statistically different from IMD (*p* < 0.05). Nevertheless, when compared to IMZ and IMD, at 10 µM, LCT produced the lowest AChE inhibition (~7%; *p* < 0.05). To the best of our knowledge, the data here presented reports for the first time on the in vitro inhibition of AChE by LCT in a system designed for extrapolation to human toxicity. Concerning other animal ecotoxicity studies, LCT was found to decrease cholinesterase activity in *Rana cyanophlyctis* (a frog species) at brain, liver and kidney level, using tissue homogenates [71]. In rats, LCT reduced the expression of AChE in the hippocampus [21]. In *Channa punctatus* (snakehead fish), brain, gills and muscle AChE activity was reduced after LCT exposure [22].

### 3.2. Pesticide-Induced Tyrosinase Inhibition

Although the most common function attributed to tyrosinase is the production of the skin pigment melanin, this enzyme is also involved in the production of neuromelanin, a pigment found in neurons and associated with neurodegeneration. However, the presence of neuromelanin has also been associated with neuroprotection against heavy metal toxicity. Among the neurons affected by neuromelanin toxicity, loss of dopaminergic neurons is correlated with Parkinson’s disease onset [30]. Nevertheless, the association between tyrosinase and Parkinson’s disease is not clear. In the skin, tyrosinase, a monophenol monooxygenase, promotes tyrosine hydroxylation and L-DOPA oxidation [72], and its role in neuromelanin synthesis is still being unveiled, although its expression in the brain and its overexpression in age-dependent neuromelanin production and in increase dopamine toxicity has been reported [30,41,72,73].

Nevertheless, none of the pesticides tested in the present research modulated tyrosinase activity; thus, it is possible to assume that any toxicity observed by these pesticides is not due to a direct effect on the activity of tyrosinase. Regardless, it cannot be excluded the potential effect of the pesticides on the protein expression, which may induce a different outcome in vivo assays.

A previous study using GLY revealed that the herbicide modulated melanin production in insect species but in a tyrosinase-independent pathway, interacting with L-DOPA oxidation [73]. Ford et al. Ford, et al. [74] analyzed the effect of various neonicotinoid insecticides on plant tyrosinase activity; none of the compounds were active against plant tyrosinase; however, some of the pesticide metabolites were. Regarding IMZ, a study using zebrafish hypothesized that this fungicide may inhibit tyrosinase as the mechanism behind loss of pigmentation, but this hypothesis was not tested [75]. LCT was able to inhibit tyrosinase activity in *Micromelalopha troglodyte*; however, no studies were reported regarding extrapolation to human toxicity [76]. We report here for the first time, to the best of our knowledge, the non-inhibitory effect of imazalil against tyrosinase.

## 4. Conclusions

Concerning the effect of toxicants, some in vitro assays may have limited extrapolation to in vivo (e.g., human exposure to pesticides). As in vivo effects may be impaired by absorption and metabolism processes, which decrease bioavailability, the effective concentrations of pesticides in target tissues are lower than the tested concentrations, making the concentration-effect correlation difficult. Thus, in vitro enzymatic inhibition assays provide a valuable tool to screen compounds for neurotoxicity dependent on key enzyme inhibition. A significant number of commonly used pesticides described to have low toxicity to animals has yet not been screened for their modulatory effect on cholinesterase-dependent neurological processes, and most studies concerning this topic are focused on ecotoxicological application. In this research, we report the comparison of AChE, BChE and tyrosinase inhibition by glyphosate, imazalil, imidacloprid and λ-cyhalothrin. It was observed that glyphosate presented the lowest neurotoxic activity, with low inhibition of AChE activity in concentrations up to 1 mM. The insecticides imidacloprid and λ-cyhalothrin present selective AChE inhibition, while the fungicide IMZ is a broad-spectrum cholinesterase inhibitor, capable of inhibiting AChE and BChE in an equal manner. None of the pesticides tested was able to modulate tyrosinase activity. While additional studies should be carried out to further understand the mechanism behind cholinesterase inhibition, we here report the use of in vitro enzyme-based methodologies to screen pesticides for their neurotoxicity.

## Data Availability

Not applicable.

## Supplementary Materials

The following supporting information can be downloaded at: https://www.mdpi.com/article/10.3390/toxics10080448/s1, Table S1. “Results for ANOVA statistical analysis”.
