# Supplementary material for: Neurotoxicity Assessment of Four Different Pesticides Using In Vitro Enzymatic Inhibition Assays
Source: Toxics. 2022 Aug 3;10(8):448. doi: 10.3390/toxics10080448 (PMC9413506; doi:10.3390/toxics10080448)
Supplement: Supplementary file 1 [file toxics-10-00448-s001.zip › toxics-1805021-supplementary.pdf]

Supplementary material

Neurotoxicity assessment of four different pesticides using *in vitro* enzymatic inhibition assays

Carlos Martins-Gomes [1,2], Tiago E. Coutinho [1,2], Tânia L. Silva [1,2], Tatiana Andreani [2,3] and Amélia M. Silva [1,2,*]

[1] Department of Biology and Environment, School of Life Sciences and Environment, University of Trás-os-Montes e Alto Douro (UTAD), Quinta de Prados; 5001-801 Vila Real, Portugal; amsilva@utad.pt (A.M.S.)
[2] Center for Research and Technology of Agro-Environmental and Biological Sciences (CITAB-UTAD), Quinta de Prados, 5001-801 Vila Real, Portugal; camgomes@utad.pt (C.M.G.), tecoutinho@utad.pt (T.E.C.); tanialfs10@gmail.com (T.L.S.); tatiana.andreani@fc.up.pt (T.A.)
[3] GreenUPorto—Sustainable Agrifood Production Research Centre & Department of Biology, Faculty of Sciences of the University of Porto, Rua do Campo Alegre s/n, 4169-007 Porto, Portugal;
[*] Correspondence: amsilva@utad.pt (A.M.S.); Tel.: +351-259-350-921 (A.M.S.)

Statistical significance of differences between samples and the control, or between different concentrations, was assessed by the analysis of variance (ANOVA) with Tukey's multiple comparison test (α = 0.05), and statistical significances between results of the two enzymes were assessed by two-way ANOVA with Sidak´s multiple comparison test (α = 0.05), using the tools of GraphPad Prism version 7 (GraphPad Software Inc., San Diego, CA, USA). Table S1, summarizes the results of the statistical analysis concerning F-value, *p*-value and degrees of freedom.

Table S1. Results for ANOVA statistical analysis.

		**ANOVA**	**F-value**	***p*-value**	**Degrees of freedom**
**Glyphosate**	AChE inhibition	1	26.35	<0.0001	6
	BChE inhibition	1	0.09598	0.9957	6
	AChE *vs* BChE	2	20.24	<0.0001	6
**Imazalil**	AChE inhibition	1	37.32	<0.0001	6
	BChE inhibition	1	366.7	<0.0001	6
	AChE *vs* BChE	2	16.24	<0.0001	6
**Imidacloprid**	AChE inhibition	1	121.9	<0.0001	6
	BChE inhibition	1	0.2449	0.9535	6
	AChE *vs* BChE	2	118.0	<0.0001	6
**λ-cyhalothrin**	AChE inhibition	1	287.0	<0.0001	6
	BChE inhibition	1	2.334	0.0898	6
	AChE *vs* BChE	2	264.2	<0.0001	6

Notes: 1 – One-way ANOVA; 2 – Two-way ANOVA
